# 肺癌患者中耶氏肺孢子菌肺炎的研究进展

**DOI:** 10.3779/j.issn.1009-3419.2022.101.14

**Published:** 2022-04-20

**Authors:** 婷 李, 建英 周, 晴 王

**Affiliations:** 310000 杭州，浙江大学医学院附属第一医院呼吸与危重症医学科 Department of Respiratory Disease, Thoracic Disease Center, The First Affiliated Hospital, College of Medicine, Zhejiang University, Hangzhou 310000, China

**Keywords:** 肺肿瘤, 耶氏肺孢子菌肺炎, 危险因素, 放化疗, 免疫检查点抑制剂, Lung neoplasms, *Pneumocystis jirovecii* pneumonia, Risk factors, Chemoradiotherapy, Immune checkpoint inhibitors

## Abstract

近年来，随着免疫抑制剂的广泛使用，耶氏肺孢子菌肺炎（*Pneumocystis jirovecii* pneumonia, PJP）在非艾滋病（如恶性肿瘤、器官移植术后及自身免疫性疾病）患者中的发病率显著升高。PJP在血液系统肿瘤及器官移植术后等人群中的危险因素及诊治得到了广泛研究，但在实体肿瘤中的研究有所欠缺。而肺癌为全球发病人数和死亡人数最高的肿瘤，临床上肺癌患者感染PJP的预后较差。本文通过回顾既往文献，总结了PJP在肺癌患者中的流行病学及临床表现，肺癌患者感染PJP的危险因素及可能机制、诊断及预防等研究进展，为临床应用提供参考。

## Abstract (en)

In recent years, with the widespread use of immunodepressant agents, *Pneumocystis jirovecii* pneumonia (PJP) has been significantly found in non-human immunodeficiency virus (HIV) patients, such as those with malignancies, post-transplantation and autoimmune diseases. Although the risk factors and management of PJP have been extensively studied in the hematologic tumor and post-transplant populations, the research on real tumor cases is insufficient. Lung cancer has been the most common tumor with the highest number of incidence and death worldwide, and the prognosis of lung cancer patients infected with PJP is poor in clinical practice. By reviewing the previous studies, this paper summarized the epidemiology and clinical manifestations of PJP in lung cancer patients, the risk factors and possible mechanisms of PJP infection in lung cancer patients, diagnosis and prevention, and other research progresses to provide reference for clinical application.

耶氏肺孢子菌肺炎（*Pneumocystis jirovecii* pneumonia, PJP），旧称卡氏肺孢子虫肺炎，是由PJ引起的一种机会性真菌感染性疾病。该疾病主要表现为发热、咳嗽及呼吸困难，严重时可出现呼吸衰竭，典型的影像学特征为双侧弥漫性的肺间质病变^[1-3]^。一般认为，PJP好发于艾滋病人群，然而随着近几十年来免疫抑制剂的广泛使用，PJP在非艾滋病（如恶性肿瘤、器官移植术后及自身免疫性疾病）患者中的发病率显著升高^[1, 4]^。

## 1 概述与流行病学

肺癌是全球发病人数和死亡人数最高的肿瘤^[5]^，法国一项1990年-2010年的回顾性研究结果^[6]^显示在肺癌人群中PJP的发病率为2.6/10万人年，肺癌患者在感染PJP的非艾滋病患者中占2%，在感染PJP的实体肿瘤患者中占10.7%。而在日本的一项2006年-2018年的回顾性研究^[7]^中，感染PJP的非艾滋病实体肿瘤患者中肺癌患者占30%。在预后方面，Lee等^[8]^的研究显示，肺癌合并PJP感染的非艾滋病患者3个月的病死率高达61.6%，提示肺癌合并PJP感染患者预后较差。尽管随着近年来诊断及治疗水平的提高，PJP的预后已有所改善^[9]^，但仍需引起我们重视。

针对不同病理类型的肺癌患者，2019年韩国一项关于112例合并PJP感染的肺癌患者的回顾性研究^[8]^显示：感染人群中肺腺癌占46.6%，鳞癌占36.6%，小细胞肺癌占12.5%，与肺癌病理类型分布基本相似；而肿瘤分期则为：Ⅲ期占18.7%，Ⅳ期占74.1%。在其他研究^[10]^中也有相似的结论，可见PJP感染多见于晚期的肺癌患者，而与肺癌的病理类型无关，这可能与下文将提到的晚期肺癌的治疗带来的PJP感染风险有关。

## 2 临床表现及影像学表现

Lee等^[8]^的研究中肺癌患者感染PJP后最常见的临床症状为呼吸困难（71.4%），其次为发热（27%）和咳嗽（23%），病情进展迅速，从诊断肺癌到诊断PJP感染的时间为197 d（25 d-1, 114 d）。该结果与感染PJP的非艾滋病患者相比，发热与咳嗽的比例较少^[11]^。而章巍等^[12]^报道的4例肺癌合并PJP的患者均出现了发热、呼吸困难及咳嗽，感染PJP距肺癌诊断的时间最短为3个月，最长为12个月。在影像学表现方面，PJP在胸部计算机断层扫描（computed tomography, CT）上的典型表现为双侧对称的弥漫性磨玻璃影。但有时放疗导致的放射性肺炎也同样表现为弥漫性肺磨玻璃浸润影，使得患者肺部影像复杂难辨，引起PJP诊断的延迟，甚至误诊，导致患者病情的加重。有研究^[13]^认为，放射性肺炎病变的范围通常与照射野一致，且多表现为蜂窝状及网状影，而PJP病灶浸润常出现在未受辐射的肺野^[14]^。这可能有助于进行鉴别，但在临床上仍需结合患者的其他临床特点综合判断，必要时行病原学检测明确诊断。有观点认为正电子发射计算机断层显像（positron emission tomography computed tomography, PET/CT）可能有助于PJP的早期诊断。Haeusler等^[15]^的研究发现PJP感染的8例肿瘤患者PET/CT均表现为双侧肺部摄取信号增强，且其中2例患者在PET/CT检查已提示异常时，胸片和肺部CT表现正常或仅有轻微病变。Kono等^[16]^的报道也提示了PET/CT能在肺部CT之前观察到肺部受累。PET/CT的常用显像剂^18^F-氟代脱氧葡萄糖除了能被肿瘤细胞摄取，也可被中性粒细胞、巨噬细胞等多种炎症细胞、病原体以及肉芽组织摄取。有研究^[17]^认为，肺组织真菌感染时出现“呼吸爆发”的现象，炎症细胞大量吞噬病原体可导致细胞葡萄糖代谢显著增加，我们推测，这可能是PET/CT能够早于肺部CT，在炎症早期即发现病灶的原因。

## 3 肺癌患者中PJP的危险因素研究

### 3.1 糖皮质激素的使用

业已证实，糖皮质激素的使用是非艾滋病患者感染PJP的独立危险因素，而在肺癌患者中，Lee等^[8]^的研究认为高剂量糖皮质激素的长期使用（等效醋酸泼尼松20 mg/d，持续 > 3周）是肺癌患者感染PJP的危险因素之一。在肺癌患者中糖皮质激素主要应用于肿瘤治疗本身，如肺癌脑转移患者、预防或治疗放化疗引起的呕吐、皮疹等副反应，放疗引起的放射性肺炎，免疫治疗引起的免疫相关性不良反应等。既往研究^[18, 19]^显示肺癌放疗患者中急性放射性肺炎的发生率超过20%，而放射性肺炎的治疗一般首选糖皮质激素。糖皮质激素的使用导致这类人群发生PJP的风险增加。

而对于免疫治疗，我们知道免疫检查点抑制剂（immune checkpoint inhibitors, ICIs）主要通过免疫阻断程序性死亡受体1（programmed cell death protein 1, PD-1）/程序性死亡配体1（programmed death ligand 1, PD-L1）和细胞毒性T淋巴细胞相关抗原4（cytotoxic T lymphocyte antigen 4, CTLA-4）检查点恢复或增强机体的抗肿瘤免疫，带来生存获益的同时也会伴有不良反应的发生，包括肺炎、肝炎、肠炎以及皮疹等^[20]^，尤其是免疫相关性肺炎的发生率在非小细胞肺癌中较高^[21]^。而2级以上不良反应的治疗往往需要糖皮质激素的使用，3级以上则需要高剂量糖皮质激素使用至少4周，甚至必要时联合免疫抑制剂。目前有观点^[22]^认为激素及免疫抑制剂的使用使得这类患者感染PJ的风险明显增加。且由于免疫介导引起的肺损伤以及肿瘤本身引起的反应，这类感染PJ的患者往往很快出现重症肺炎甚至呼吸衰竭。Del Castillo等^[23]^的研究显示740例使用免疫治疗的黑色素瘤患者发生严重感染（包括PJP）的危险因素之一即为糖皮质激素的使用。而在肺癌患者中目前仅有个案报道，Schwarz等^[24]^报告了2例非小细胞肺癌患者使用免疫抑制剂联合大剂量糖皮质激素治疗PD-L1抑制剂Nivolumb引起的免疫相关性肺炎，最后患者感染PJP并死于呼吸衰竭。

### 3.2 放疗和化疗

既往研究^[25]^表明，肿瘤患者的化疗和/或放疗是发生PJP的高危因素。在血液系统肿瘤中，一些特定的化疗药物如氟达拉滨会增加PJ感染的风险。而在肺癌患者中，Lee等^[8]^关于肺癌治疗与PJP感染相关性的研究认为高剂量糖皮质激素的长期使用和同步放化疗是肺癌患者发生PJP的危险因素。该研究中肺癌患者诊断PJP时的化疗时间中位数为97 d，涉及的化疗药物涉及铂类、吉西他滨、多西他赛、紫杉醇等，但研究^[8]^认为感染PJP的风险与具体的化疗药物种类无关；该研究中诊断PJP时的放疗累积剂量中位数为54 Gy；另一项研究^[7]^中，5例确诊PJP的肺癌患者有4例未曾使用中-高剂量糖皮质激素，但发病前均进行化疗和肺部放疗（具体化疗方案：紫杉醇联合顺铂或卡铂；放疗累积剂量：39 Gy-66 Gy）。而在北爱尔兰2011年-2016年所有接受根治性放疗的683例肺癌患者中，有14例最终死于PJP，死亡时平均累积剂量为55 Gy，放疗时间为3个月^[26]^。由此可见，放疗、放疗联合化疗是肺癌患者感染PJP的危险因素，可能涉及的机制有：①使得肿瘤患者糖皮质激素的应用增加：预防及治疗放化疗引起的呕吐、皮疹、放射性肺炎等副反应；②放化疗对免疫功能的抑制：尤其引起患者淋巴细胞数目减少、骨髓抑制等。Mcaleese等^[26]^的研究中14例接受放疗最终死于PJP的肺癌患者中有13例出现持续超过1个月的低淋巴细胞血症，入院时淋巴细胞计数中位数为0.4×10^9^/L，低于0.6×10^9^/L。而淋巴细胞对辐射较敏感，放疗可引起患者的CD4^+^ T淋巴细胞以及CD8^+^ T淋巴细胞凋亡^[27]^。而化疗方面，吉西他滨常见的副反应为中性粒细胞数目减少和骨髓抑制，Lingaratnam等^[28]^的研究中9例涉及吉西他滨化疗的PJP患者中有7例均存在低淋巴细胞血症，淋巴细胞计数范围为0.35×10^9^/L-0.86×10^9^/L，这可能导致吉西他滨与PJP感染相关。另一项研究^[29]^中以多西他赛为基础的化疗患者感染PJP时均出现CD4^+^ T淋巴细胞数目减少（0.2×10^4^/L）；③放化疗引起肺呼吸道黏膜的损伤。这些因素使得接受放化疗的肺癌患者PJP感染风险明显增加。

### 3.3 免疫治疗

近些年来，随着肿瘤靶向治疗及免疫治疗的发展和使用，肺癌的治疗方法呈多样化，患者的预期寿命得到延长。Lee等^[8]^的研究中确诊PJP的肺癌患者组中使用免疫治疗占9.4%，与没有诊断PJP的肺癌组相比没有差异。在免疫治疗中，鉴于ICIs对免疫的促进作用，如促进T细胞启动及活性，目前主要观点认为ICIs本身不会增加患者感染PJ的可能性^[22, 30]^。相反地，Zhang等^[31]^的研究认为使用PD-L1抑制剂能促进小鼠体内PJ的清除。他们的研究发现在动物实验中，相对于正常的小鼠，存在*PD-1*基因缺陷的小鼠和使用抗PD-1抗体的小鼠感染PJP后肺泡巨噬细胞的吞噬功能增强，同时，肺组织中的辅助型T细胞（T helper cell, Th）1/Th17型免疫反应亦有明显增强，从固有免疫及适应性免疫两方面均促进了病原体的清除，因此检测到的PJ数量更少^[31]^。有研究^[32, 33]^发现骨髓源性抑制细胞可通过激活PD-1/PD-L1途径抑制巨噬细胞的吞噬作用，从而导致机体清除PJP能力下降。而抗PD-1/PD-L1抗体能够通过阻断PD-1途径，恢复巨噬细胞的吞噬功能，促进PJP的清除。抗PD-1抗体对病原体的清除作用在其他真菌研究中也有相似结论，包括隐球菌^[34]^、荚膜组织胞浆菌^[35]^等。但如上文所说，当免疫治疗过程中产生不良反应时，如免疫相关性肺炎、肝炎等，激素及免疫抑制剂的使用反而可能使这类人群患PJP的风险增加。

## 4 肺癌患者中PJ定植与感染

鉴于PJP在肺癌患者中病情进展快，致死率高，PJP的早期发现与诊断有利于及时的抗感染治疗，改善预后。相对于传统方法的对PJ包囊或者滋养体的检测，实时荧光定量聚合酶链反应（quantitative real-time polymerase chain reaction, qPCR）以及宏基因组二代测序（metagenomics next-generation sequencing, mNGS）等基因技术检测PJ的敏感性更高，但无法鉴别是PJ定植还是感染^[36]^。PJ定植的定义为可检测到患者肺部存在PJ病原体，但无PJP的典型临床表现或影像学表现^[37, 38]^。有研究^[38]^使用qPCR检测196例非艾滋病患者的肺泡灌洗液标本，结合临床表现最后诊断PJP 36例（18.3%），定植33例（16.8%）。但在肺癌患者中，目前尚无涉及PJ定植率的大样本研究。一项前瞻性研究^[39]^检测35例肺癌患者的肺泡灌洗液发现有6例患者的样本存在PJ定植，且研究认为糖皮质激素是定植的独立危险因素。Togashi等^[40]^对47例无PJP临床表现的晚期肺癌患者的呼吸道标本进行PJ PCR检测，结果全为阴性。而另一项研究^[41]^发现206例肺癌患者在最初诊断肺癌时PJP定植达到5.8%。这些检测结果的不同可能因取样部位、取样方式、取样时所处的疾病阶段以及地域不同导致。虽然已有研究^[42]^表明可以根据肺泡灌洗液qPCR检测的拷贝数确定阳性感染和阴性的界定值（分别为31, 600拷贝/mL和3, 160拷贝/mL），但两者中间仍存在很大的灰色地带。Kang等^[41]^的研究认为肺癌患者气道中的微生物定植与后期患者肺炎发生无关，包括PJ。这与早期Togashi对晚期肺癌PJ的定植与发生的研究观点^[40]^相似。这提示我们提前对肺癌患者气道进行PJ定植的检测对预测后续PJP的发生并无帮助^[41]^。最终PJP的诊断仍需在病原体检测的基础上结合临床表现及肺部影像学表现以最终明确。

## 5 PJP的预防

有研究^[43, 44]^发现，接受药物磺胺甲恶唑-甲氧苄啶（TMP-SMX）预防的非艾滋病患者相较于未预防者，PJP的发生率可降低85%-91%，同时相关死亡率也显著下降。基于TMP-SMX已证实的有效性，推荐将其作为PJP预防的一线用药^[45, 46]^。一般认为，预防性用药适用于PJP发病率高以及用药收益超过风险（如毒副作用）的人群，如器官移植术后、CD4^+^细胞计数持续 < 200个/μL、持续较大剂量使用糖皮质激素（醋酸泼尼松 > 20 mg/d，持续超过4周）、严重营养不良等人群^[44]^。鉴于肺癌患者PJP的患病率并不高，不推荐对一般的肺癌患者进行常规药物预防。但当肺癌患者存在以下高危因素：①长期使用大剂量糖皮质激素；②进行放疗和/或化疗，尤其出现低淋巴细胞血症时，使用药物预防PJP的发生是必要的^[47, 48]^。对于免疫治疗，有专家建议当接受PD-1/PD-L1抑制剂和CTLA-4抑制剂治疗的非艾滋病患者采用20 mg/d醋酸泼尼松或等效糖皮质激素治疗超过4周时应进行PJP的预防^[30]^。

## 6 总结

肺癌患者中PJP的患病率不高，但往往病情重，致死率高，对于存在高危因素如放疗和/或化疗、长期大剂量激素使用的肺癌患者，尤其在出现低淋巴细胞血症时推荐进行药物预防。当存在高危因素的肺癌患者出现典型临床表现及影像学表现时，我们需警惕PJ感染的可能性，实现早期诊断及治疗。
